# Transposable Elements Re-Wire and Fine-Tune the Transcriptome

**DOI:** 10.1371/journal.pgen.1003234

**Published:** 2013-01-24

**Authors:** Michael Cowley, Rebecca J. Oakey

**Affiliations:** Department of Medical & Molecular Genetics, King's College London, London, United Kingdom; University College London, United Kingdom

## Abstract

What good are transposable elements (TEs)? Although their activity can be harmful to host genomes and can cause disease, they nevertheless represent an important source of genetic variation that has helped shape genomes. In this review, we examine the impact of TEs, collectively referred to as the mobilome, on the transcriptome. We explore how TEs—particularly retrotransposons—contribute to transcript diversity and consider their potential significance as a source of small RNAs that regulate host gene transcription. We also discuss a critical role for the mobilome in engineering transcriptional networks, permitting coordinated gene expression, and facilitating the evolution of novel physiological processes.

## Introduction

The 1983 Nobel Prize for Physiology or Medicine was awarded to Barbara McClintock for her seminal discovery of transposable elements (TEs). McClintock's studies of colour patterns in maize kernels led her to conclude that “controlling elements” that could jump around the genome regulate gene expression (reviewed in [1]). Although the response of the scientific community to her work was initially cautious, the discovery of similar elements in flies, bacteria, and yeast underlined its significance. Today, TEs are recognised as important components of genomes that have helped shape their evolution.

**Figure 1:**
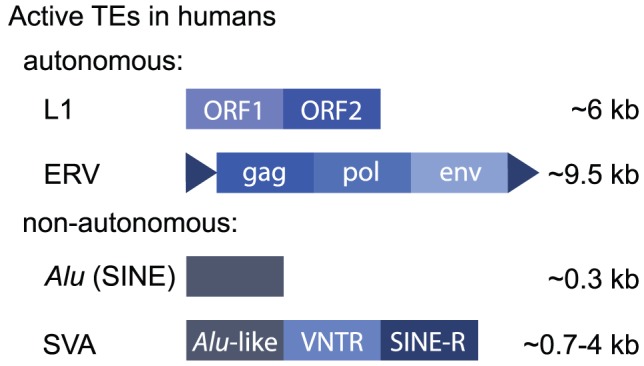
Active human retrotransposons.

Approximately half of the human genome is derived from TEs [2], although recent work suggests this may be closer to two-thirds [3]. Most human TEs are *retrotransposons*, and some are still active today (Box 1 and Figure 1). Consequently, TEs represent a significant source of genetic variation [4]–[7].

**Box 1. The Human Mobilome**

Human TEs may be classified as retrotransposons, which replicate using an mRNA intermediate to “copy and paste,” or DNA transposons, which transpose using a DNA intermediate. Retrotransposons constitute ∼42% of the human genome [2], and some elements are still active today, meaning they are still capable of retrotransposing. DNA transposons represent ∼3% of the human genome but are no longer transposition-competent. Retrotransposons can be further classified based on their structure. LTR elements are characterised by long terminal repeats and include endogenous retroviruses (ERVs) that encode *gag*, *pol*, and *env* genes. These evolved as a consequence of retroviral infection of germ cells, so that they are inherited through generations (reviewed in [77]). Other LTRs are “solo” LTRs, meaning they exist alone. These result from a recombination event that deletes the intervening retroviral genes [78]. Non-LTR retrotransposons include long and short interspersed repeat elements (LINEs and SINEs) and SVA elements that are a composite of sequences derived from other repeats (SINE, VNTR [variable number tandem repeat], and *Alu*). There are only three highly active elements in the human genome: (1) a subset of L1 LINEs, (2) *Alu* elements that are a family of primate-specific SINEs, and (3) SVA elements (Figure 1). *Alu* elements are the most active, with approximately one de novo germline insertion per 20 births [79]. De novo insertions of L1 and SVA elements occur at the rate of approximately 1 in 108 births and 1 in 916 births, respectively [4], [80]. Some ERVs may still be active in humans [81], although the vast majority are nonfunctional, in contrast to their relatively high levels of activity in mice. Only L1 elements encode the enzymes required for retrotransposition, and these preferentially (but not exclusively) recognise L1 mRNA molecules. *Alu* and SVA elements co-opt the L1 machinery to retrotranspose. The mechanism of retrotransposition has been expertly reviewed elsewhere recently [82], [83].

**Figure 2:**
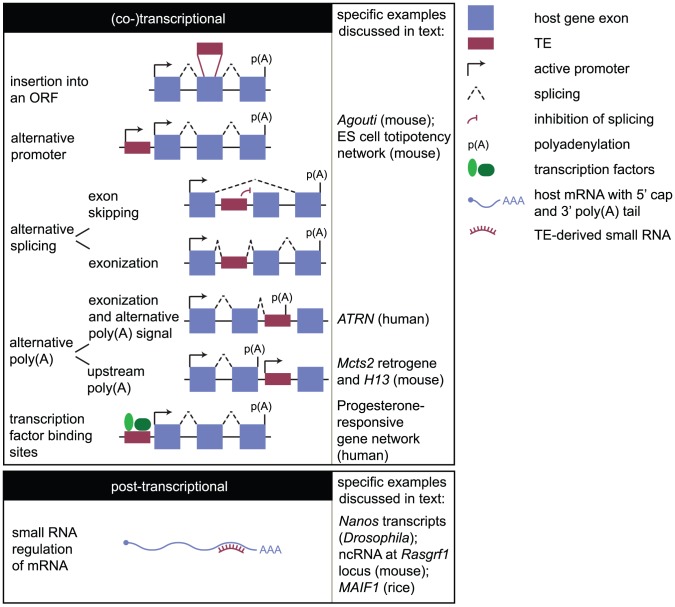
How the mobilome can impact the transcriptome. Impacts on the transcriptome may be considered transcriptional (or co-transcriptional) and posttranscriptional. The former mechanisms include insertion of a TE into an ORF; provision of an alternative promoter that may be tissue- or stage-specific in its activity; promotion of alternative splicing either through prevention of the splicing machinery from recognising a splice acceptor site in an endogenous exon (exon skipping) or through incorporation of the TE into the mature transcript (exonization); promotion of alternative polyadenylation (poly(A)) either by providing an alternative polyadenylation signal or by promoter activity interfering with host gene transcription and causing upstream polyadenylation; and by introducing transcription factor binding sites that may confer tissue- or stage-specific expression, or link a gene into a transcriptional network. Posttranscriptional regulation involves TE-derived small RNAs binding to host transcripts. In the case of *Drosophila Nanos* transcripts, small RNAs destabilise the transcript by recruiting the deadenylation machinery. In the case of murine *Rasgrf1*, the binding of small RNAs to an ncRNA associated with one allele results in the recruitment of the de novo methylation machinery to that allele, causing allele-specific *Rasgrf1* expression. The events occurring downstream of small RNA binding are therefore diverse and locus-specific.

How might TEs influence gene expression? It is easy to imagine how an insertion into a gene might disrupt an open reading frame (ORF), preventing the synthesis of a protein (Figure 2). Indeed, examples of human diseases caused in this manner have been reported [8], [9]. However, the impact of an insertion may not be so dramatic or deleterious. TEs can influence host genes by providing novel promoters, splice sites, or polyadenylation signals (Figure 2). An important consequence is the generation of transcript diversity. There are many more different mRNA molecules in the human transcriptome than the 20,000 protein-coding genes in the genome, and this transcript diversity is thought to be key for promoting phenotypic diversity in higher eukaryotes [10], [11]. Additionally, genome-scale studies have revealed the importance of TEs in dispersing transcription factor binding sites, linking genes in transcriptional networks (e.g., [12], [13]), and facilitating the evolution of novel traits.

In this review, we consider how TEs, collectively referred to as the *mobilome*, have impacted the transcriptome. This includes elements active today as well as those no longer transposition-competent. Although not nearly an exhaustive account, we draw on specific examples from a range of organisms to illustrate the variety of mechanisms through which this can occur. We aim to highlight the importance of the mobilome in shaping both the diversity and regulation of the transcriptome.

## Generating Transcriptome Diversity

One surprising finding from sequencing the human genome was that humans have a similar number of genes to the model nematode *Caenorhabditis elegans*: about 20,000 and 19,000, respectively. This was unexpected because of the apparent complexity of humans relative to nematodes; indeed, earlier estimates for the number of human genes ranged from 60,000–150,000. Several factors may account for this discrepancy [14], but one important consideration is alternative mRNA processing. This includes alternative splicing and polyadenylation, enabling multiple mRNA species to be generated from a single gene. These mRNA isoforms can encode proteins with different functions or may be differentially regulated. More than 95% of human multi-exonic genes are alternatively spliced [15], while this is around 25% in *C. elegans* [16].

TEs, particularly L1 and *Alu* elements, can introduce novel splice sites [17], [18]. Indeed, *Alu* elements inserted into a gene can provide both splice acceptor and donor sites, creating new exons [19], [20]. Moreover, most *Alu*-derived exons are alternatively spliced [21], contributing to transcript diversity. They are enriched in the 5′ untranslated regions of human genes, where they regulate mRNA translation [22]. Furthermore, many alternative splicing events of *Alu*-derived exons are tissue-specific, suggesting TEs contribute to the transcriptome differences that define cell types [23]–[25]. Polyadenylation stabilises mRNA transcripts and influences nuclear export and translation efficiency. The majority of human genes utilise alternative polyadenylation sites [26], and the signals for some of these are embedded in TEs [27], suggesting TEs can influence the 3′ end processing of host gene transcripts.

**Figure 3:**
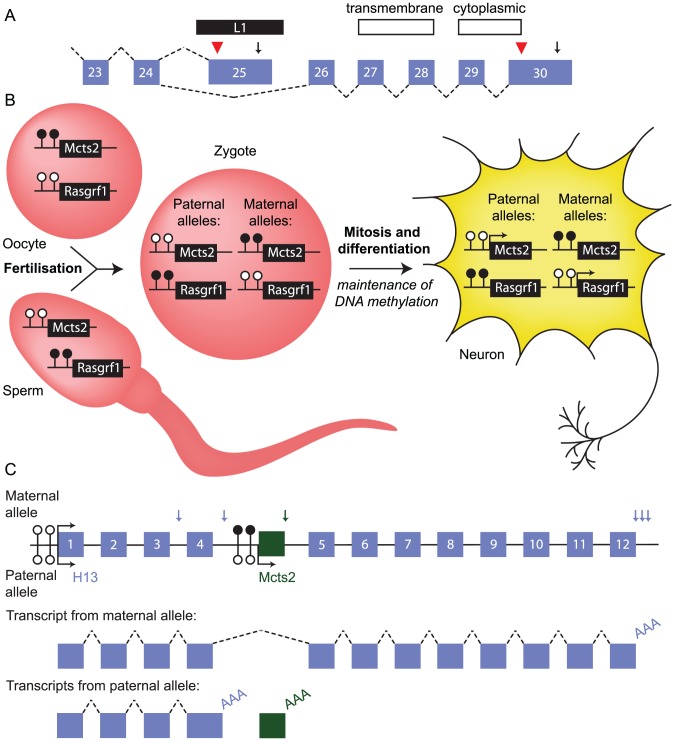
Retrotransposition can influence mRNA processing. (A) Schematic of the 3′ end of the human *ATRN* gene. An L1 element (black bar) inserted between exons 24 and 26 (numbered boxes) provides a terminal exon, translation termination site (red arrowhead), and polyadenylation signal (arrow) for a subset of transcripts. Alternative splicing produces an mRNA isoform that is polyadenylated in exon 30; only this isoform encodes transmembrane and cytoplasmic domains. Dashed lines, splicing event. (B) Inheritance of DNA methylation at the imprinted *Mcts2* and *Rasgrf1* genes in mouse. The promoter of *Mcts2* is methylated (filled lollipops) in the oocyte and unmethylated (empty lollipops) in sperm. This is opposite to the *Rasgrf1* promoter. After fertilisation, these differences persist, marking the origin of the parental alleles even in terminally differentiated cell types, where the unmethylated promoters are transcriptionally active (arrows). (C) Relationship between the retrogene *Mcts2* and the gene *H13*. (Top) Locus structure. *Mcts2* (green box) is situated between exons 4 and 5 of *H13*. Allele-specific differences in methylation at the *Mcts2* promoter result in expression of *Mcts2* from the paternal allele only. The *H13* promoter is unmethylated and active on both alleles. *H13* transcripts use alternative polyadenylation sites (vertical blue arrows). Vertical green arrow, single *Mcts2* polyadenylation site. (Middle) Representative transcript produced from transcription of the maternal allele. *H13* transcripts splice around *Mcts2* and use one of three downstream polyadenylation signals (one transcript is shown for clarity). (Bottom) Representative transcripts produced from transcription of the paternal allele. *Mcts2* is transcribed and the mRNA is polyadenylated (AAA). *H13* transcripts use one of two upstream polyadenylation signals (one transcript is shown for clarity). Transcription of the retrogene *Mcts2* is associated with upstream polyadenylation of *H13* transcripts.

The human *ATRN* gene provides a good example of how TE-induced alternative mRNA processing can enable functional diversification of one gene. A subset of *ATRN* transcripts are cleaved and polyadenylated within an L1 element that has retrotransposed into an intron (Figure 3A) [28]. Other transcripts splice around the L1 element and incorporate an additional five exons. Transcripts polyadenylated within the L1 element encode a soluble form of Attractin that is released by activated T lymphocytes as part of the basic inflammatory response [29]. The alternative transcripts encode a protein with transmembrane and cytoplasmic domains that is membrane-bound. This isoform is similar to murine Atrn, which functions as a receptor involved in pigmentation and energy metabolism [28], [30], [31]. This is a clear example of how a single retrotransposition event can increase transcript diversity with direct consequences on cellular function.

mRNA processing can also be impacted by TEs indirectly. Although the L1-encoded retrotransposition enzymes preferentially recognise L1 mRNA molecules, host protein-coding mRNAs can also be retrotransposed by the L1 machinery, creating a copy of the original gene [32]. In most cases the copy is nonfunctional, but it can potentially evolve into a retrogene with a novel function or expression pattern. About 120 retroposed sequences have evolved into bona fide genes in the human genome [33]. Retrogenes embedded in introns of other genes can influence transcription of that gene, causing upstream transcript polyadenylation. This is a mechanism through which TEs can indirectly influence mRNA processing, further promoting transcript diversity.

In some cases, the impact of the retrogene on host mRNA processing is specific to only one of the two parental alleles [34], [35]. For example, the retrogene *Mcts2* is embedded in an intron of *H13*. *Mcts2* is subject to genomic imprinting. It is expressed exclusively from the allele inherited through the paternal line because its promoter is silenced on the maternally inherited allele by DNA methylation (Figure 3B). Silencing of *Mcts2* on the maternally inherited allele permits transcription of *H13* to continue through the retrogene, which is spliced out of mature transcripts, and downstream polyadenylation sites are used (Figure 3C). *Mcts2* transcription on the paternally inherited allele is associated with *H13* transcripts using upstream polyadenylation sites. This is not a consequence of introducing alternative polyadenylation signals, but may involve the elongation complexes that are transcribing *H13* “crashing” into those at the transcribing retrogene, a process termed “transcriptional interference” [36]. This interference may promote *H13* transcript cleavage and polyadenylation. Intronic ERVs may influence transcription in a similar manner at many loci, impacting on the levels of protein produced from the endogenous gene [37], [38].

TEs further promote transcript diversity by providing alternative promoters for host genes. Perhaps one of the most elegant examples of this can be found in viable yellow agouti (*A^vy^*) mice, in which an ERV intracisternal A particle (IAP) upstream of the *Agouti* gene provides an alternative promoter than can drive ectopic *Agouti* expression, producing yellow fur [39]. Although the overt phenotype means this is a well-studied specific example, high-throughput approaches have demonstrated widespread use of TEs as alternative gene promoters in normal tissues, and these contribute to tissue-specific expression profiles [24], [40]. Inappropriate activation of promoters embedded in TEs, perhaps resulting from the relaxation of repressive epigenetic marks, can drive ectopic gene expression, and this mechanism has been implicated in human diseases, including cancer [24], [41].

These examples illustrate the impacts of retrotransposition events that occurred in the germline and are stably inherited. Recently there has been much debate about the extent and significance of somatic retrotransposition. L1 expression increases as neural stem cells commit to a neuronal lineage, and this was reported to be associated with elevated L1 retrotransposition [42]. The proposed outcome of this is increased transcriptome heterogeneity among neurons, contributing to interindividual variation [43]. However, the true extent of this mechanism in vivo is debated, with recent estimates ranging from ∼80 to fewer than 0.6 somatic L1 insertions per neuron in the human brain [44], [45], depending on the experimental approach used. Additional work will be required to confirm the true significance of this mechanism for promoting somatic transcriptome diversity. However, the generation of transcript diversity by alternative mRNA processing and the utilisation of alternative promoters in the genome inherited through the germline is likely to be a key factor in the evolution of higher eukaryotes. The mobilome has played a significant role in this process, both directly, by introducing regulatory sequences, and indirectly, by interfering with host gene transcription.

## The Mobilome as a Source of Small Regulatory RNAs

*Mcts2* is one example of ∼150 genes that are subject to genomic imprinting in the mouse. Although the mechanisms for regulating imprinting vary between loci, silencing of one allele by DNA methylation is a common theme. DNA methylation is likely to have evolved initially as a host defence mechanism against TE expression. Indeed, male mouse germ cells lacking the de novo methyltransferase *Dnmt3L* exhibit elevated expression of L1 elements and IAPs, resulting in meiotic catastrophe [46]. Thus, the need to minimise the impacts of retrotransposons may have driven the evolution of a novel mode of gene regulation—genomic imprinting—that is critical for mammalian development ([47] and reviewed in [48], [49]).

In addition to DNA methylation, small regulatory RNAs, including PIWI-interacting RNAs (piRNAs) and small interfering RNAs (siRNAs), may also have evolved as a host defence mechanism, repressing the translation of TE transcripts or promoting their decay. Like DNA methylation, this mechanism has been adopted by the host to regulate endogenous genes. Indeed, TEs are major players in the production of small RNAs that regulate host gene transcripts. For example, TE-encoded piRNAs are required to establish a gradient of maternal *Nanos* mRNA transcripts in the early *Drosophila* embryo [50]. This is achieved by the binding of piRNAs to a specific sequence in the 3′ untranslated region of *Nanos* transcripts, which promotes removal of the polyadenylate tail and transcript degradation. This process is essential for establishing correct anterior-posterior patterning in the embryo.

Mammals also utilise TE-encoded small RNAs to regulate host gene expression. In mice, the imprinted gene *Rasgrf1*, like *Mcts2*, is under the control of differential DNA methylation on the two alleles. At this locus, DNA methylation is established in the paternal germline (Figure 3B), opposite to that for *Mcts2*, and this requires TE-encoded piRNAs [51]. During spermatogenesis, these piRNAs bind to a noncoding (nc)RNA transcribed from the locus; specifically, they recognise an LTR-type retrotransposon RMER4B embedded within the ncRNA. Targeting of this ncRNA results in recruitment of the de novo methylation machinery, through an unknown mechanism. Disruption of the piRNA pathway or expression of the ncRNA perturbs methylation and imprinting of *Rasgrf1*, and mice defective for *Rasgrf1* imprinting exhibit impaired postnatal growth [52].

These examples from *Drosophila* and mice demonstrate the importance of TEs as a source of small RNAs for regulating host transcripts. As such, the host depends upon TEs to provide these regulatory molecules, illustrating their intimate relationship. This relationship is not exclusive to animals, with plants utilising the same system to fine-tune gene expression. For example, in rice, siRNAs originating from the miniature inverted-repeat TE (MITE) *Stowaway1* regulate tolerance to abiotic stress [53]. These siRNAs may function by targeting transcripts of the growth regulator *MAIF1* and stalling growth, a common physiological response in plants to abiotic stresses.

A defensive response to TEs is critical to guard against uncontrolled transposition. Hosts have evolved several mechanisms for tackling this, including transcriptional repression by DNA methylation and posttranscriptional repression by small RNAs. The evolution of these systems has dramatically impacted the transcriptome because they have been adopted for more general gene regulation.

## TEs as Engineers of Transcriptional Networks

TEs can influence alternative mRNA processing or generate small regulatory RNAs, but these effects on the transcriptome are locus-specific. How is transcription regulated on a global scale, say in response to an environmental cue? In the yeast *Saccharomyces cerevisiae*, genes involved in common metabolic pathways are physically clustered in the genome [54], [55], permitting co-ordinated expression [56] and tight gene regulation in response to a stimulus. However, not all genes that must be co-ordinately expressed are physically clustered. In higher eukaryotes, the binding of transcription factors can co-ordinately activate the expression of genes dispersed throughout the genome. Genes linked in this manner can be considered part of a single transcriptional network. The mobilome has been vital for linking genes in this manner. Regulatory elements required for TE expression can be co-opted by endogenous genes, or the TE may harbour transcription factor binding sites (TFBSs; Figure 2) [12], [13], [57], [58]. TE-derived TFBSs evolve rapidly relative to non-repeat-derived sites [59], suggesting they are important drivers in conferring species-specific gene expression profiles.

A good example of the importance of TEs in linking genes in a network can be found in embryonic stem (ES) cells. ES cells are pluripotent but can enter a transient phase of totipotency from which they can generate both embryonic and extra-embryonic lineages [60]. This switch depends on the activation of a network of transcripts that initiate within ERV LTRs and is controlled by epigenetic modifications. In the pluripotent state, ERVs are transcriptionally repressed, in part by histone 3 lysine 9 methylation [61]. This is established by the histone methyltransferase SETDB1 that is recruited to ERVs by KAP1 [62], [63]. ES cells deficient for *Kap1* switch more readily to the totipotent state, consistent with the idea that relaxation of ERV repression drives network activation [60]. These studies highlight a critical role for ERVs in contributing to host cell fate decisions by activating a transcriptional network. This is mediated by epigenetic marks that are established and removed by endogenous cellular machinery.

Earlier, we discussed how a TE could contribute to the evolution of novel functions at a single gene, such as human *ATRN*. However, by re-wiring networks, the mobilome can facilitate the evolution of complex physiological processes involving gene expression on a global scale. The evolution of pregnancy, the trait that defines mammals, is an intriguing example of this. The hormone progesterone triggers the differentiation of endometrial stromal cells to form the decidua, the maternal component of the placenta [64]. This relies on the activation of a network of transcripts linked by MER20 elements that provide binding sites for progesterone-responsive signalling molecules [65].

An important progesterone-responsive gene is prolactin. In addition to being linked in this network by MER20, the promoter for human prolactin is derived from an independent TE, MER39 [66]. This TE is primate-specific, yet other mammals activate prolactin expression during pregnancy. Emera et al. [67] demonstrated that the endometrial stromal cell-specific promoters of human, mouse, and elephant prolactin are all distinct and are all derived from different TEs (MER77 for mouse, L1 for elephant), suggesting TEs can contribute to convergent evolution. Similarly, the *syncytin* genes, essential for formation of the syncytiotrophoblast layer that mediates fetal-maternal exchange, are derived from ERV *env* genes and have been independently acquired in the human, mouse, and rabbit genomes [68].

Other aspects of the physiological changes required for pregnancy may have evolved by TEs re-wiring networks, such as the tolerance of the maternal immune system to a fetus expressing paternal antigens [69]. Together, these examples illustrate the requirement for TEs in pregnancy: engineering a transcriptional network, providing cell type-specific promoters, and contributing to gene function. Thus, the impact of TEs can extend well beyond single-locus effects, making vital contributions to the evolution of complex physiological processes. This role is not confined to animals; TEs in plants have had similar impacts [70].

## Conclusions and Future Perspectives

The impacts of TEs on the host transcriptome are diverse. At the single locus level, a transposition event may result in an alternatively processed transcript that can evolve a new function. At the genome level, TEs may disperse regulatory elements that rewire transcriptional networks. The significance of TEs is becoming increasingly apparent with more widespread application of next-generation sequencing technologies. At first, repetitive elements were a nuisance in the analysis of genome-wide datasets; now new experimental protocols and computational pipelines are being utilised to ask questions specifically about TE distribution [71], [72]. The 1000 Genomes Project will provide a valuable tool for interrogating the extent and functional importance of insertional polymorphisms in humans, and indeed has already yielded some intriguing findings; for example, the insertion rates of TEs differ between populations [73].

Many important questions remain unanswered. For example, what is the extent and biological significance of somatic retrotransposition? What is the contribution of this mechanism to the transcriptome differences between neurons, and how does this influence behaviour? Additionally, inappropriate activation of TEs has been associated with somatic cancers [71]. It is too early to say if this mechanism is truly causative, but the current data are provocative. Another exciting area with important outstanding questions is the influence of epigenetic silencing of TEs on the host. For example, could methylated TEs act as “messengers,” transmitting epigenetic information between generations? During primordial germ cell development, most DNA methylation is erased and reset, but a small fraction of the genome is resistant to erasure. This includes IAPs, suggesting these are candidates for mediating transgenerational epigenetic inheritance [74], [75]. Indeed, such a role has been demonstrated for at least two specific IAPs [39], [76], but whether this represents a more global mechanism is undetermined.

Are the evolutionary benefits conferred by TEs purely accidental? Most point mutations arising in the germline have a deleterious or neutral effect on the host, but some do introduce innovative changes that are beneficial. Likewise, TE insertions may be deleterious but can also provide opportunities for increasing transcript diversity or rewiring the transcriptome. Such advantageous insertion events can be selected for and fixed in a population. This “fine-tuning” of the transcriptome could explain why organisms have evolved mechanisms to regulate TE activity without completely silencing all types.

Our understanding of the diverse impacts of the mobilome on the transcriptome has come a long way since the finding that TEs could cause insertional mutations leading to disease. TEs have been fundamental players in evolution and are intimately associated with the regulation of host gene transcription.
